# Neoadjuvant Hormonal Therapy is Associated with Comparable Outcomes to Neoadjuvant Chemotherapy in Post-Menopausal Women with Estrogen Receptor-Positive Breast Cancer

**DOI:** 10.3389/fonc.2013.00317

**Published:** 2013-12-27

**Authors:** David M. Marcus, Jeffrey M. Switchenko, Roshan Prabhu, Ruth O’Regan, Amelia Zelnak, Carolina Fasola, Donna Mister, Mylin A. Torres

**Affiliations:** ^1^Department of Radiation Oncology, Emory University, Atlanta, GA, USA; ^2^Winship Cancer Institute, Emory University, Atlanta, GA, USA; ^3^Department of Biostatistics and Bioinformatics, Emory University, Atlanta, GA, USA; ^4^Department of Hematology and Oncology, Emory University, Atlanta, GA, USA

**Keywords:** breast cancer, neoadjuvant therapy, chemotherapy, hormonal therapy, outcomes

## Abstract

**Objectives:** We compared outcomes in post-menopausal estrogen receptor-positive (ER+) breast cancer patients treated with neoadjuvant hormonal therapy (NAHT) or neoadjuvant chemotherapy (NACT).

**Methods:** We retrospectively identified post-menopausal women who received either NAHT or NACT for non-metastatic, non-inflammatory, ER+, Her2neu negative breast cancer from 2004 to 2011. We compared long-term rates of locoregional relapse free survival (LRFS), distant metastasis free survival (DMFS), and overall survival (OS) using the Kaplan–Meier method. The Cox proportional hazards model was used to identify patient and disease factors significantly associated with these endpoints.

**Results:** We identified 99 patients in our study, including 27 who received NAHT and 72 who received NACT. There were no differences in 4-year LRFS, DMFS, or OS between groups. On Cox proportional hazards modeling, the type of systemic therapy (NAHT versus NACT) was not associated with OS. However, patients with progesterone receptor (PR) positive disease had a 92% lower risk of death compared to patients with PR negative disease.

**Conclusion:** Our data suggest that outcomes are not adversely affected by NAHT in post-menopausal women with ER+ breast cancer. Therefore, NAHT is a viable and potentially less toxic option than NACT in appropriately selected patients. Furthermore, although PR negative disease appears to be associated with poor prognosis, intensification of systemic treatment with chemotherapy may not be associated with improvement of disease-related outcomes in this patient population.

## Introduction

For patients with invasive cancers of the breast, both chemotherapy and hormonal therapy are important components of definitive treatment. Chemotherapy has been historically recommended for breast cancer patients with tumors greater than 1.0 cm in size and/or lymph node positive disease. Based on several randomized studies demonstrating a significant survival benefit in women treated with tamoxifen, hormonal therapy is considered an additional component of standard therapy in women with hormone receptor-positive disease (1).

Traditionally, both chemotherapy and hormonal therapy have been given in the adjuvant setting. However, since the publication of the National Surgical Adjuvant Breast and Bowel Project (NSABP) B-18 trial, chemotherapy has been increasingly administered before surgery in women with breast cancer (2). There are several advantages to sequencing systemic therapy in this manner. First, neoadjuvant chemotherapy (NACT) may facilitate surgery in initially inoperable patients. Second, NACT may increase the likelihood of breast conserving surgery (BCS) in patients presenting with large tumors. Third, the use of NACT enables clinicians to directly observe the degree of tumor response to a specific chemotherapy regimen, which may both guide further treatment and provide prognostic information. Lastly, administering systemic therapy, rather than surgery, initially may provide a theoretical opportunity to eradicate microscopic circulating tumor cells quickly and potentially prevent development of distant metastasis in high risk patients.

The response of a tumor to NACT is largely dependent on the systemic agents used and the tumor’s receptor status. Several studies have demonstrated that “triple-negative” tumors [i.e., tumors that lack expression of estrogen receptor (ER), progesterone receptor (PR), and Her2neu] are more likely to exhibit a pathologic complete response (pCR) to NACT compared to ER and PR positive tumors (3, 4). Furthermore, of all molecular subtypes, tumors expressing Her2neu have been shown to have the highest rates of pCR, particularly when Her2neu-directed therapies are included with their chemotherapy regimens (5). Based on these findings, consensus guidelines have included low or absent hormone receptor status as a factor in patient selection for NACT (2, 6).

Contrary to triple-negative and Her2neu positive tumors, breast tumors expressing ER and/or PR are frequently less sensitive to chemotherapy than other tumor subtypes (7). Furthermore, having less than a pCR to NACT in patients with hormone receptor-positive disease may not share the same prognostic significance as it does in women with triple-negative or Her2neu positive tumors (4). Due to the high proportion of ER and/or PR positive tumors that exhibit little response to NACT, many clinicians question the benefit of chemotherapy in patients with hormone receptor-positive disease. Furthermore, studies validating the use of a 21-gene recurrence score assay (OncotypeDx) support the notion that up to 50% of hormone receptor-positive patients will not benefit from chemotherapy regardless of tumor size and nodal status (8–10). However, recognizing the benefits of upfront systemic therapy, many physicians are increasingly prescribing neoadjuvant hormonal therapy (NAHT) to women with hormone receptor-positive breast cancer as an alternative to NACT, particularly in those who have co-morbidities precluding chemotherapy treatment.

Nevertheless, prospective clinical data on the use of NAHT in invasive breast cancer is scarce, and there are few studies comparing NAHT and NACT in patients with estrogen receptor-positive (ER+) breast cancers. The goal of our study was to compare long-term outcomes of post-menopausal breast cancer patients with ER+ tumors treated with NAHT versus those treated with NACT followed by surgery.

## Materials and Methods

After obtaining approval from the Emory institutional review board, we identified post-menopausal patients treated with NAHT or NACT for non-metastatic, non-inflammatory, ER+, invasive breast cancer at Emory University between 2004 and 2011. Patients with tumors expressing Her2neu were excluded. Age greater than 50 was used as a surrogate for post-menopausal status in women whose menopausal status was not recorded in the medical record. None of the NAHT patients underwent adjuvant chemotherapy. Patients who received systemic therapy but did not ultimately undergo surgical resection of their tumor were excluded.

The delivery, type, dose, and duration of NAHT or NACT were determined by the treating medical oncologist. Relevant patient, tumor, and treatment characteristics were collected from the medical record including patient age at diagnosis, tumor size (both before and after neoadjuvant therapy), presence or absence of pCR (defined as the absence of any invasive cancer in the breast and lymph nodes in the pathologic specimen) (11), PR status, tumor grade, lymphovascular space invasion, type of NAHT [tamoxifen versus aromatase inhibitor (AI)], type of NACT (anthracycline-based versus non-anthracycline-based), use of adjuvant radiation therapy (XRT), and type of surgery (mastectomy versus BCS). Locoregional recurrence free survival (LRFS), distant metastasis free survival (DMFS), and overall survival (OS) were determined. LRFS was defined as time to either locoregional recurrence (including failure in the ipsilateral breast, chest wall, or axillary, supraclavicular, or internal mammary lymph nodes) or death, and DMFS was defined as time to either distant metastasis or death. Time to all endpoints was calculated from the date of pathologic diagnosis of breast cancer.

All statistics were calculated using SAS Software, Version 9.3 (SAS, Cary, NC, USA). Pearson’s Chi-squared test was used to determine whether differences in proportions existed between groups with regard to baseline patient characteristics, unless expected cell counts were less than 5, in which case Fisher’s Exact test was used. The two-sample *t*-test was used to compare means of continuous variables. The Wilcoxon test was used to compare median values for continuous variables. Kaplan–Meier curves were generated to evaluate survival, and time-to-event data between groups was compared by the log-rank test. Cox proportional hazards modeling was used to identify important patient and disease factors associated with primary and secondary endpoints. A *p*-value of ≤0.05 was considered to be statistically significant.

## Results

### Patient characteristics

Of the 99 patients included in the study, 72 (72.7%) received NACT, and 27 (27.3%) received NAHT. Median age of all patients was 59.0 years. As shown in Table 1, patients receiving NAHT were older than patients receiving NACT (mean age 67.9 versus 58.0 years, *p* < 0.01), and patients receiving NAHT had lower grade tumors (*p* = 0.03) and less advanced N stage (*p* < 0.01) compared to patients receiving NACT.

**Table 1:** **Baseline patient characteristics for all patients receiving either neoadjuvant chemotherapy (NACT) or neoadjuvant hormone therapy (NAHT) prior to surgery for estrogen receptor-positive breast cancer at Emory University between 2004 and 2011**.

	NACT		NAHT		*p*-Value*
	*n*	%	*n*	%	
T stage					0.29
T1	12	16.9	8	30.8	
T2	33	46.8	13	50.0	
T3	22	31.0	5	19.2	
T4	4	5.6	0	0.0	
N stage					<0.01
N0	20	29.9	17	63.0	
N1	33	49.3	4	14.8	
N2	11	16.4	4	14.8	
N3	3	4.5	2	7.4	
PR status					0.31
Positive	30	62.5	20	74.1	
Negative	18	37.5	7	25.9	
Tumor grade					0.03
Grade 1	14	24.6	4	19.1	
Grade 2	23	40.4	15	71.4	
Grade 3	20	35.1	2	9.5	
Type of carcinoma					0.84
Ductal	40	62.5	18	66.7	
Lobular	22	34.4	9	33.3	
Mixed	1	1.6	0	0.0	
Other	1	1.6	0	0.0	
Adjuvant XRT					0.19
Yes	55	80.9	17	68.0	
No	13	19.1	8	32.0	
Mean age	58.0		67.9		<0.01

*PR, progesterone receptor; XRT, radiation therapy*.

*p-Values represent chi-squared test for all categorical variables and *t-*test for comparison of means of continuous variables.

*p-Value calculated with chi-square test or Fisher’s Exact test where appropriate for categorical variables. Two-sample *t*-test used for comparisons of mean age.

### Treatment

Among the NACT patients, the majority (50.8%) received both an anthracycline and a taxane, while 38.1% received an anthracycline-based regimen alone. Of patients receiving NAHT, 92.6% received an AI, and 7.4% received tamoxifen. The median duration of NAHT was 8.0 months (range 0.5–60.0 months).

### Outcomes

Median follow-up for all patients was 45.7 months (range 2.8–138.7 months). Very few patients developed a pCR. In fact, all patients in the NAHT group were found to have residual disease at the time of surgery, while in the NACT group, six patients (6.9%) achieved a pCR. There was no statistically significant difference in pCR rates between the two groups (*p* = 0.32). Mean tumor shrinkage was 22.8% for patients receiving NACT and 23.0% for patients receiving NAHT (*p* = 0.99). Rates of pathologic response (either pCR or pathologic partial response) were 68.1% for NACT patients and 80.0% for NAHT patients (*p* = 0.28). There was no significant difference in median NAHT duration between NAHT patients with pathologic response compared to those with no pathologic response (7.5 versus 13.0 months, *p* = 0.57). Among patients receiving NACT, rates of pathologic response were not higher for patients receiving both an anthracycline and a taxane (pathologic response seen in 71.4% of patients) compared to patients receiving either an anthracycline alone (75.0%) or a taxane alone (25.0%) (*p* = 0.19).

**Figure 1:**
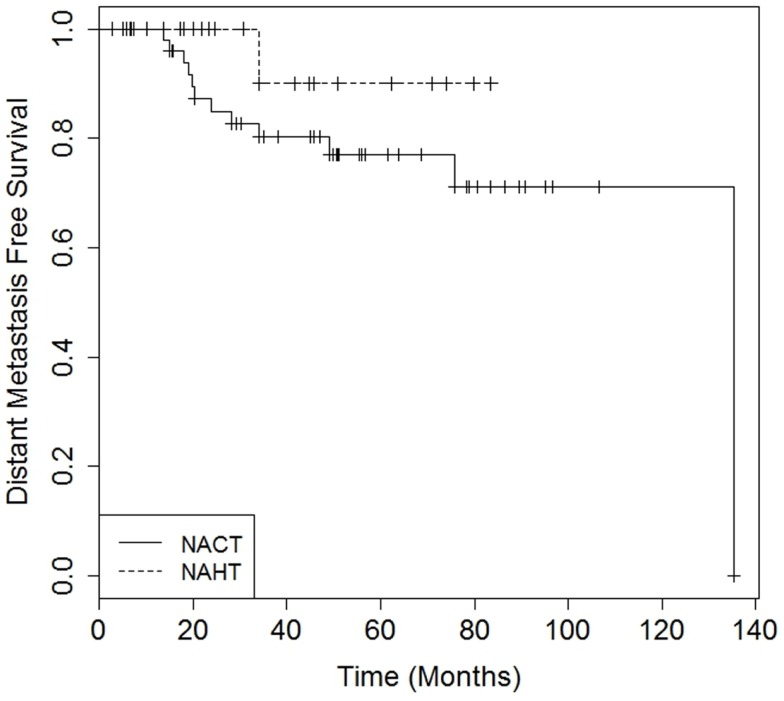
**Kaplan–Meier curves illustrating distant metastasis free survival over time for patients receiving neoadjuvant hormonal therapy (NAHT) versus neoadjuvant chemotherapy (NACT)**. Log-rank *p*-value = 0.41.

**Figure 2:**
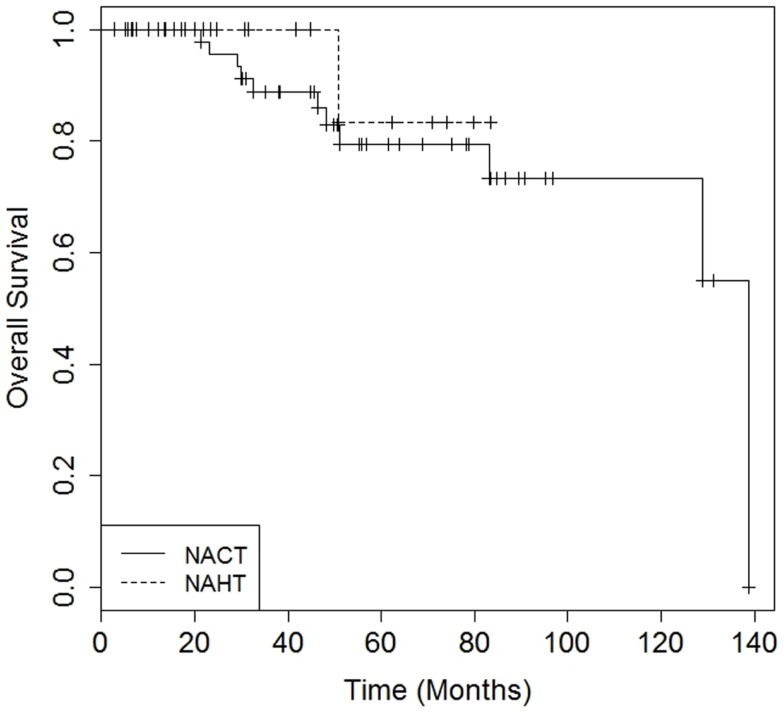
**Kaplan–Meier curves illustrating overall survival over time for patients receiving neoadjuvant hormonal therapy (NAHT) versus neoadjuvant chemotherapy (NACT)**. Log rank*p*-value = 0.49.

There were no differences in DMFS (4-year DMFS 90.0% for NAHT versus 80.3% for NACT, *p* = 0.41) or OS (4-year OS 100.0% for NAHT versus 85.9% for NACT, *p* = 0.49) between NAHT and NACT patients (Figures 1 and 2). Cox proportional hazards modeling identified PR status (HR = 0.08, 95% CI 0.01–0.68, *p* = 0.02) as a significant predictor of OS, while advanced T stage (*p* = 0.96), advanced N stage (*p* = 0.07), tumor grade (*p* = 0.09), and neoadjuvant therapy type (NACT versus NAHT, *p* = 0.50) were not significant factors for OS in the analysis. Risk of death was 92% lower in the patients with PR positive disease compared to those with PR negative disease. There were no significant differences in LRFS between NAHT and NACT patients (*p* = 0.40). None of the NAHT patients recurred locally, while two NACT patients treated with mastectomy and no XRT developed locoregional recurrence. Given the low overall event rate for local recurrence, distant recurrence, and death, multivariate analysis was unable to be performed.

## Discussion

Findings from our study support the use of NAHT in select post-menopausal breast cancer patients with ER+ disease. In this patient cohort, PR negativity was a predictor of poor outcome, but tumor grade, N stage, patient age, and the type of neoadjuvant systemic therapy (NAHT versus NACT) were not. Although NACT patients were younger and had more advanced stage disease, there were no differences in LRFS, DMFS, or OS between patients treated with NAHT and NACT, suggesting that NACT may potentially mitigate the effects of these risk factors on outcome. While NACT appears warranted in these patients with high risk disease, NAHT alone may be sufficient in older women with less advanced ER+ breast cancer.

As noted previously, there is very little available data comparing long-term outcomes in women treated with modern NAHT and NACT. Moreover, the majority of published data on NAHT alone pertains to patients who are either unable to tolerate chemotherapy or have a short life expectancy that precludes chemotherapeutic treatment. While pCR rates associated with NAHT are generally low, our study and other research suggest that NAHT may be an effective therapy in appropriately selected patients. In one prospective Phase II study involving 94 breast cancer patients with operable breast tumors measuring at least 4 cm, all patients received NAHT, with NACT reserved for tumors that failed to respond to NAHT and for tumors not expressing ER. This study was not limited to post-menopausal patients with ER+ disease, and the majority of NACT patients were treated with cyclophosphamide, doxorubicin, vincristine, and prednisolone, a chemotherapy regimen no longer used in standard practice. Nevertheless, pCR rates were similar to what we found in our cohort with none of the NAHT patients and only eight NACT patients achieving a pCR. In spite of the relatively low pathologic response rate, there was no difference in OS between patients who received NAHT alone and those who received NACT after a median follow-up period of 7.5 years (12). Additionally, a randomized phase II study from Russia demonstrated that NAHT was associated with rates of objective response and BCS that were comparable to those of NACT in 121 post-menopausal patients with ER+ or PR+ breast cancers (13). However, this study did not evaluate local control, rates of distant metastasis, or survival, and to date there is remarkably little data describing whether NACT and NAHT are associated with comparable results in terms of disease outcomes in this patient population.

Given the paucity of data comparing the use of NAHT to NACT for hormone sensitive breast cancer and outcome, the results of our study may help guide decision-making regarding neoadjuvant therapy in post-menopausal breast cancer patients with ER+ disease. The primary limitation of our study is its retrospective nature, which potentially introduces a selection bias in terms of the type of neoadjuvant therapy delivered. The small numbers of patients and limited follow-up also preclude definitive conclusions, but our data (along with the other small studies noted previously) support the notion that NAHT may be an effective alternative to NACT. Furthermore, although the type of NAHT was fairly homogenous, the duration of therapy varied widely, which may have had an effect on the efficacy of the treatment (although NAHT duration did not appear to influence outcome). Within the cohort who received NACT, there was also variability in the type of chemotherapy delivered, with 50.8% of patients receiving both an anthracycline and a taxane and 38.1% of patients receiving an anthracycline alone. Finally, although the majority of baseline patient characteristics were balanced between groups, patients receiving NACT had more advanced N stage and higher grade tumors compared to patients receiving NAHT, which is consistent with the current practice of delivering NACT in patients with aggressive disease. These imbalances between treatment groups indicate possible selection bias, and the variability in the type and duration of systemic therapy within the groups may further preclude definitive conclusions about the efficacy of NAHT versus NACT. Based on these limitations, in addition to the small size of the cohort and limited follow-up, our results must be interpreted with caution. Nonetheless, the findings of our study are provocative and merit further exploration, ideally in the setting of a prospective randomized trial.

The results of our study demonstrate encouraging effectiveness of NAHT, with no differences in LRFS, DMFS, or OS found between patients receiving NAHT and those receiving NACT. Furthermore, while PR negativity appears to be associated with poor prognosis, intensification of systemic treatment may not be associated with improvement of disease-related outcomes in this patient population. Although prospective data is needed to confirm these results, our data suggest that NAHT is a viable treatment option for appropriately selected post-menopausal patients with ER+ breast cancer.

## Conflict of Interest Statement

The authors declare that the research was conducted in the absence of any commercial or financial relationships that could be construed as a potential conflict of interest.
